# Seminal and vagino-uterine microbiome and their individual and interactive effects on cattle fertility

**DOI:** 10.3389/fmicb.2022.1029128

**Published:** 2022-11-08

**Authors:** Sarah M. Luecke, Emily M. Webb, Carl R. Dahlen, Lawrence P. Reynolds, Samat Amat

**Affiliations:** ^1^Department of Microbiological Sciences, North Dakota State University, Fargo, ND, United States; ^2^Department of Animal Sciences, and Center for Nutrition and Pregnancy, North Dakota State University, Fargo, ND, United States

**Keywords:** reproductive tract microbiome, bovine, interactive effects, semen, fertility, reproductive health

## Abstract

Reproductive failure is a major economical drain on cow-calf operations across the globe. It can occur in both males and females and stem from prenatal and postnatal influences. Therefore, the cattle industry has been making efforts to improve fertility and the pregnancy rate in cattle herds as an attempt to maintain sustainability and profitability of cattle production. Despite the advancements made in genetic selection, nutrition, and the implementation of various reproductive technologies, fertility rates have not significantly improved in the past 50 years. This signifies a missing factor or factors in current reproductive management practices that influence successful fertilization and pregnancy. Emerging lines of evidence derived from human and other animals including cattle suggest that the microbial continuum along the male and female reproductive tracts are associated with male and female fertility—that is, fertilization, implantation, and pregnancy success—highlighting the potential for harnessing the male and female reproductive microbiome to improve fertility in cattle. The objective of this narrative review is to provide an overview of the recent studies on the bovine seminal and vagino-uterine microbiome and discuss individual and interactive roles of these microbial communities in defining cattle fertility.

## Introduction

One of the most fundamental aspects of successful and profitable beef and dairy cattle operations is maintaining a strong reproductive efficiency within the herd. Advancements in reproductive technology that have been made in the past few decades have been profound, providing producers with Expected Progeny Differences (EPDs) to assist with genetic selection, estrous synchronization to pair with artificial insemination, and even cryopreservation of gametes from genetically superior animals (Dahlen et al., 2014). In addition, genetic improvement of fertility through the application of advanced genomic and proteomics technologies combined with bioinformatics tools is increasing to enhance cattle reproductive performance (Veerkamp and Beerda, 2007; Kovac et al., 2013; Crowe et al., 2018; Cai et al., 2019). There is also significant progress that has been made in the improvement of nutritional strategies to improve reproduction in cattle in recent years (Butler, 2014; Crowe et al., 2018). Despite the advances in reproductive technologies, genetic selection, and nutritional strategies, reproductive failure remains to be one of the major challenges affecting the beef cattle industry in the U.S and globally. A recent meta-analysis revealed that reproductive failure rates in beef cattle are not dissimilar today to rates from the first scientific reports made in the late 1970’s (Ayalon, 1978; Reese et al., 2020). Approximately 48% of beef cows will fail to become pregnant during the first 30 days following a single insemination and another 6% are estimated to experience pregnancy loss during the ensuing gestation (Reese et al., 2020). This statistic is concerning, especially because reproductive capability is a major limitation to the profitability of beef herds. The capital and labor invested into developing and maintaining female cattle is significantly high, and a cow must produce approximately six calves to recover the expenses associated with her upbringing and upkeep before she becomes profitable (Moorey and Biase, 2020). Unfortunately, an estimated one third of cows are likely to be removed from herds due to reproductive failure (Moorey and Biase, 2020). Thus, these statistics highlight the need for novel approaches to improving cattle reproductive efficiency.

While a multitude of management strategies that involve overall health, nutrition, and environment certainly play a role in reproductive outcomes, the microbial community (known as microbiota) residing within the male and female reproductive tracts has been less characterized as a variable that may influence reproductive efficiency in cattle. Emerging evidence derived from human and vertebrate animals suggest that the reproductive microbiome is important for reproductive health and fertility (Koedooder et al., 2019b; Rowe et al., 2020; Tomaiuolo et al., 2020; Ong et al., 2021). The associations of the vaginal and uterine (vagino-uterine) microbiota with fertility, implantation, and preterm birth are increasingly known (Hyman et al., 2014; Moreno et al., 2016; Benner et al., 2018; Koedooder et al., 2019b; Zhao et al., 2020). Recent developments point out that the microbiome-mediated female fertility is not only influenced by the microbial community residing within her own genital tract, but may also be affected by the microbes-associated with semen that are introduced into the vagina during intercourse (Mändar et al., 2015; Altmäe, 2018; Schoenmakers et al., 2019). The interactions between the seminal microbiota and the microbial community and immune system of the female upper reproductive tract may influence endometrium receptivity and preimplantation embryo dynamics (Robertson and Sharkey, 2016), and thereby aid conception success (Koedooder et al., 2019a). In addition to its influence on female reproductive microbiome and fertility, emerging evidence derived from humans (Weng et al., 2014; Baud et al., 2019; Okwelogu et al., 2021), horses (Quiñones-Pérez et al., 2021), and cattle (Koziol et al., 2022) suggest that the seminal microbiota may also be important for sperm development and male fertility. Accordingly, the reproductive microbiome holds potential to offer novel opportunities for reducing the incidence of reproductive failures in cattle. To harness the microbiome for improved cattle fertility, it is of critical importance to understand taxonomic and functional properties of the microbial community residing within the male and female reproductive tract, their evolution and development, factors shaping these microbial communities, and microbial sources. Given that fertilization and implantation could be influenced by the so called “temporary combined male and female microbiome” (Koedooder et al., 2019a), it is important to understand the interactions between seminal and vagino-uterine microbiome, and their interactive effects on female fertility. Building on the information available from reproductive microbiome-related research in the human medical and obstetric fields, in the current narrative review, we provide an overview of the reproductive microbiome in both male and female cattle and discuss the potential role of seminal and vagino-uterine microbiome as independent and interactive contributors to reproductive health and fertility. We also discuss challenges associated with studying the bovine reproductive microbiome, along with the future opportunities that reproductive microbiome-targeted research may provide to enhance fertility in cattle. Considering the emerging insights into potential involvement of the microbiome in Developmental Origin of Health and Disease (DOHaD; Stiemsma and Michels, 2018; Codagnone et al., 2019; Amat et al., 2022), and *in utero* microbial colonization (Al Alam et al., 2020; Guzman et al., 2020; Rackaityte et al., 2020; Amat et al., 2021a), we also highlight the role of the female reproductive microbiome extending beyond the reproductive performance. Of note, the literature search for this narrative review was conducted using PubMed and Google Scholar and by reviewing in-text citations of included publications. The search was limited to publications written in English. Inclusion of literature was based on the availability of information regarding the bovine male or female reproductive microbiota, other vertebrates, or human reproductive microbiome that we felt was relevant to the theme and discussion of this review.

## Male reproductive tract microbiome

### Seminal microbiota and its potential involvement in male fertility

Historically, the presence of any microbial agents in the male reproductive tract has been associated with infection, and the microorganisms identified were often regarded as pathogenic (Rowe et al., 2021). However, this ideology has recently been challenged by the identification of the presence of a commensal microbial community in the semen of healthy individuals *via* culture-independent, high-throughput sequencing (Weng et al., 2014; Tomaiuolo et al., 2020). According to a comprehensive review, human semen harbors a relatively rich and complex microbial community, and is dominated by bacterial phyla including Actinobacteria, Bacteroides, Firmicutes, and Proteobacteria (Hou et al., 2013; Chen et al., 2018; Brandão et al., 2021). In addition to the high-throughput sequencing, the Gram staining of human semen samples revealed that there was a greater number of bacterial cells than the total number of sperm cells present in the semen, and these bacterial cells exhibited diverse cell morphologies, supporting the sequencing results and indicating the high microbial community diversity in the semen (Hou et al., 2013). While the taxonomic composition of the seminal microbiota is relatively well documented, there is limited information available on functional properties of this microbial community. A recent systemic review based on a meta-analysis of 24 studies conducted between 1992 and 2019 revealed a link between certain bacterial species present in the semen of humans with sperm quality and motility (Farahani et al., 2021). For example, *Enterococcus faecalis* and *Corynebacteria* were associated with decreased sperm motility. An increased prevalence of *Pseudomonas* has been reported in the patients with *Oligoasthenospermia*, which is a condition in which sperm concentrations are low (Fainberg and Kashanian, 2019). *Prevotella* and *Anaerococcus* were associated with poor sperm quality. Farahani et al. (2021) also found a higher prevalence of *Ureaplasma urealyticum* in infertile men along with a negative association with sperm morphology and concentration (Farahani et al., 2021). However, *Lactobacillus* has been reported to be associated with improved semen quality (Farahani et al., 2021) and male fertility (Brandão et al., 2021), suggesting that *Lactobacillus* spp. may be important in sperm development and (or) male fertility. In addition, Koedooder and colleagues conducted a narrative review of the microbiota composition of both the male and female reproductive tracts (Koedooder et al., 2019a). They identified three seminal bacterial community clusters in the men’s semen, with each cluster being dominated by either *Lactobacillus*, *Pseudomonas*, or *Prevotella*, and also detected a correlation between *Mycoplasma* spp. abundance and reduced sperm concentration and abnormal sperm morphology (Koedooder et al., 2019a). Thus, the studies discussed above together suggest that there is self-sustaining and diverse microbial community present in the male reproductive tract, and that the seminal microbiota may play a role in sperm metabolism and development, and male fertility.

### Bovine seminal microbiota

With the increased appreciation of the seminal microbiota and its potential association with sperm development and male fertility, bovine seminal microbiota has recently begun to be explored using high-throughput sequencing (Table 1). Koziol and colleagues characterized the seminal microbiota by performing 16S rRNA gene sequencing on the semen samples of 45 beef bulls. In this study, semen samples were categorized as satisfactory (*n* = 32) or unsatisfactory (*n* = 13) after breeding soundness exams (BSE) and sperm analysis (Koziol et al., 2022). They identified a relatively rich and complex microbial community in those semen samples, and this microbial community contained facultative anaerobic and strict anaerobic bacteria and archaea. The most abundant genera identified include *Escherichia*, *Bacteroides*, *Corynebacterium*, and *Gemella* (Table 1). Those bulls with a satisfactory sperm classification (based on spermiograms that evaluated morphology and motility) had a higher relative abundance of *Escherichia coli* and *Streptococcus* species compared with those with an unsatisfactory sperm classification. Conversely, bulls with an unsatisfactory sperm classification had a greater relative abundance of *Enterococcus* species and the uncultured bacterium, S5-A14a, compared to the satisfactory bulls (Koziol et al., 2022). During sample collection, the breeds, ages, and locations of these bulls were recorded to further understand the correlation between bacterial presence in semen and other factors. Bulls in most of the farms had seminal microbial communities dominated by *Escherichia*, *Corynebacterium*, and *Bacteriodes* species. In some farms, however, bull semen harbored *Histophilus* spp., which includes pathogenic species associated with bovine respiratory disease (BRD; Griffin et al., 2010). In contrast, semen samples of bulls raised in some farms were found to have *Lactobacillus* species, which are commonly used as a probiotic. There was no significant difference in terms of alpha diversity and bacterial community structure of seminal microbiota between differing breeds or ages (Koziol et al., 2022). Strengths to this study include the categorization of samples according to breeding soundness exams performed on fresh sperm samples, as this is a frequently used indicator of bull fertility. However, there are emerging molecular markers of fertility such as seminal plasma proteins and metabolites that may also be considered in future studies (Klein et al., 2022). An additional limitation to note is a lack environmental contamination control, which is an important factor to include in microbiome studies.

**Table 1:** Taxonomic identification of male reproductive tract-associated microbiota from several recent high-throughput sequencing-based studies.

**Species population**	**Country of origin**	**Samples processed for**	**Collected samples**	**Sample collection method**	**Phylum reported**	**Genera reported**	**Key findings**	**References**
45 Beef bovine bulls of various breeds [12 months–6 years of age]	United States	16S rRNA gene sequencing (V4); Illumina MiSeq	Satisfactory spermiogram	Electroejaculation and collection using a sterile cryogenic tube	Actinobacteria, Bacteroidetes, Euryarchaeota, Firmicutes, Fusobacteria, Proteobacteria	*Escherichia-Shigella*, *Bacteroides*, *Corynebacterium_1*, *Streptococcus*, and *Histophilus*, *Gemella*	Bulls with satisfactory spermiograms had a numerically higher abundance of Bacteroides, S5-A14a, Trueperella, Methanosphaera, and Methanobrevibacter Bulls with unsatisfactory spermiograms were reported to have a numerically higher abundance of Veillonellaceae, Campylobacter, and Methanobacterium Both satisfactory and unsatisfactory semen samples showed significant microbial interactions within their sample set	Koziol et al. (2022)
			Unsatisfactory spermiogram		Proteobacteria, Firmicutes, Bacteroides, and Actinobacteria	*S5-A14a*, *Bacteroides*, *Escherishia-Shigella*, *Gemella*, *Enterococcus*, and *Histophilus*		
92 Beef bovine bulls of various breeds [15 months–9 years of age]	United States	16S rRNA gene sequencing (V4); Illumina MiSeq	Epithelial surface of the prepuce and penis	Electroejaculation with aseptic swab collection	Firmicutes, Fusobacteria, Bacteriodetes, Proteobacteria, and Actinobacteria	*Fusobacterium*, *Histophilus*, *Porphytomonas*, and *Streptobacillus*	Two community types were identified: one with low community diversity, one with high community diversity Identified genera are commonly found in soil, cow vagina, the respiratory tract, and in feces The genus Bradyrhizobium was only found in low diversity samples	Wickware et al. (2020)
18 Holstein bovine bulls of known fertility [ages 3–10 years of age]	Sweden	16S rRNA gene sequencing (V3-V4); Illumina MiSeq	Semen with positive or negative fertility correlation	Bulls were cleaned by brushing and sample was collected into a sterilized graduated collection tube using a sterilized artificial vagina	Not reported	*Porphyromonas*, *Fusobacterium*, *Ruminococcaceae UCG-010*, *Fastidiosipila*, *Ruminococcaceae UCG-005*, *Cutibacterium*, *Histophilus*, *Oceanivirga*, *Corynebacterium 1*, *Campylobacter*, *W5053*, *Dyella*, *Staphylococcus*, *Lawsonella*, *Helcococcus*, *Bacteroides*, *Capnocytophaga*, *Curvibacter*, *Kingella*, *and Enhydrobacter*	Observed a negative fertility correlation between Curvibacter, Rikenellaceae, RC9-gut-group, Cutibacterium, Ruminococcaceae UCG-005, Ruminococcaceae UCG-010, Staphylococcus, and Dyella spp. The genera W5053 and Lawsonella were enriched in low fertility bulls	Cojkic et al. (2021)
55 Holstein Friesian bovine bulls	Slovakia	16S rRNA gene sequencing (V4); Illumina MiSeq	Semen	External genitalia washed and sample was collected using a sterilized artificial vagina	Firmicutes, Proteobacteria, Fusobacteria, Acinobacteria, and Bacteroides	*Fusobacterium*, *Actinobacillus*, *Bacteroides*, *Cutibacterium*, *Staphylococcus*, and *Prevotella*	The microbiome composition could be categorized into two distinct clusters Cluster 1 contained high prevalence of Actinobacteria and Firmicutes, Cluster 2 contained high prevalence of Fusobacteria	Medo et al. (2021)
Five Equine stallions [7–17 years of age]	Sweden	Bruker Biotyper MALDI-TOF	Semen	Samples collected using a sterilized artificial vagina with a disposable liner and filter and sterilized collecting flask	Not reported	*Staphylococcus*, *Bacillus*, *Corynebacterium*, *Micrococcus*, *Fusobacterium*, *Mycoplasma*, *Pantoea*, *Brevibacillus*, *Paenibacillus*, *Actinetobacter*, and *Aerococcus*	Half of the bacterial taxa that were isolated originate from the mucus membranes and the skin *Staphylococcus* spp. was identified in all samples and *Micrococcus* spp. were identified in all the extended samples as well as in the extender The frequency of detection of bacteria was variable among the individuals, and effects of the identified bacterial on sperm quality were undetermined	Al-Kass et al. (2020)
Six Murciano-Granadina goat bucks [age not reported]	Spain	16S rRNA gene sequencing (V3-V4); Illumina MiSeq	Semen during breeding season	Samples collected using an artificial vagina, transferred into 0.25 ml plastic straws, and stored in a freezer at −80°C until analysis	Firmicutes, Proteobacteria, Fusobacteria, Actinobacteria, and Bacteroidetes	*Ureaplasma*, *Oceanivirga*, *Mannheimia*, *Fastidiosipila*, and *Sphingomonas*	The most dominant genus during the breeding and non-breeding season was *Ureaplasma* Goat semen microbiota does vary between seasons and stayed stable for 7 days within a season *Spingomonas* and *Faecalibacterium* could potentially act as biomarkers for semen quality in goat bucks	Mocé et al. (2022)
			Semen during non-breeding season		Firmicutes, Proteobacteria, Fusobacteria, Actinobacteria, Bacteroidetes, and Cyanobacteria	*Ureaplasma*, *Lactobacillus*, *Bradyrhizobium*, *Chloroplast*, *Clostridium_sensu_stricto_1*, and *Romboutsia*		
38 Crossbred Bulls [1 year of age]	United States	16S rRNA gene sequencing (V3-V4); Illumina NovaSeq 6000	Semen prior to breeding	Samples collected into single use semen collection bags using electroejaculation	Fusobacteria, Bacteroidetes, Firmicutes, and Actinobacteria	*Fusobacterium*, *Prophyromonas*, *Oceanivirga*, and *Corynebacterium*	The microbial richness and diversity of semen samples increased after the 28-day breeding period Seminal microbiota was cultured in both aerobic and anaerobic conditions Seminal microbiota was unaffected by a moderate vs. a high rate of gain dietary treatment	Webb et al. (2022)
			Semen after a 28-day breeding period		Fusobacteria, Bacteroidetes, Firmicutes, and Actinobacteria	*Fusobacterium*, *Prophyromonas*, *Oceanivirga*, and *Corynebacterium*		
Six Crossbred Bulls [3–9 years of age]	Australia	Oxford Nanopore Technologies long-read adaptive sampling	Preputial mucosa and penile surface	Samples collected using a Tricamper device, transferred into PBS and stored chilled until analysis	Firmicutes, Proteobacteria, Ascomycota, and Tenericutes	Taxonomic identification done on the species level	*Histophilus somni*, *Clostridium botulinum*, and *Mycoplasmopsis californica* were the most abundant species present in the preputial samples	Ong et al. (2022)

Our group recently characterized seminal microbiota in yearling beef bulls subjected to different rates of weight gain using 16S rRNA gene sequencing, qPCR, and culturing (Webb et al., 2022; Table 1). In this study, semen samples were collected on days 0 and 112 of dietary intervention (*n* = 38 per time point) as well as post-breeding (*n* = 12). Overall, our results indicate that there is a rich and complex microbiota present in yearling beef bulls. Fusobacteriota (36.3%), Bacteroidota (30.4%), Firmicutes (17.1%), and Actinobacteriota (14.9%) were identified as the major phyla. Our qPCR results showed that the overall mean total bacterial concentrations in the semen samples collected at d 112 and post-breeding was estimated to be about 1.1 billion 16S rRNA gene copies per ml semen (Webb et al., 2022). The mean sperm cell concentration from these semen samples was 30 million per ml semen which was estimated by computer-assisted sperm analysis (CASA; Crouse et al. unpublished data). This indicates the sperm and bacteria cell ratio is about 1:3 in the semen of yearling beef bulls. Using aerobic and anaerobic culturing, we were able to isolate and identify a total of 364 bacterial isolates from the semen samples and they represented 49 different genera within the Firmicutes (60%), Proteobacteria (25%) and Actinobacteriota (9%), Bacteroidota (4%), and Fusobacteriota (2%) phyla. The most prevalent genera identified with both culturing methods were *Bacillus*, *Staphylococcus*, *Escherichia*, *Enterococcus*, and *Arthrobacter* (Webb et al., 2022).

In addition to beef bulls, microbial composition and community structure of dairy bull semen has recently been characterized. Medo and colleagues characterized the seminal microbiota of 55 Holstein-Friesian bulls using 16S rRNA gene sequencing (Medo et al., 2021; Table 1). These authors also identified two distinct microbial clusters which were enriched either by phyla Actinobacteria and Firmicutes or by Fusobacteria. The most relatively abundant bacterial genera present in the dairy bull semen were *Fusobacterium*, *Actinobacillus*, *Bacteroides*, *Cutibacerium*, *Staphylococcus*, and *Prevotella* (Medo et al., 2021). Likewise, a research team in Sweden reported that Holstein bulls (3–10 years old) harbored greater abundance of seminal bacterial genera *Porphyromonas*, *Fusobacterium*, and *Ruminococcaceae* (Cojkic et al., 2021). All combined, these sequencing-based studies revealed that bovine semen is colonized by a relatively rich and diverse microbial community, with Firmicutes, Proteobacteria, Fusobacteria, Actinobacteria, and Bacteroides being the most abundant bacterial phyla.

### Source and factors shaping bovine seminal microbiota

The male bovine reproductive tract is made up of several key tissues that are involved in either the production, transport, or delivery of seminal components and thus, may also supply the seminal microbiota. These tissues include the testis, epididymis, ductus deferens, vesicular glands, prostate gland, bulbourethral gland, the penile shaft, and glans penis (Senger, 2003). Only a few of these anatomical locations have been investigated as sources of the seminal microbiota (Alfano et al., 2018; Wickware et al., 2020). Currently, data in the literature from human reproductive studies suggest that the accessory sex glands (vesicular glands, prostate gland, and bulbourethral gland) could be one of the potential sources for seeding the seminal microbiota (Osadchiy et al., 2020; Tomaiuolo et al., 2020; Lundy et al., 2021). As such, it is highly likely that some of the microbial cells present in bovine semen originated from the accessory sex glands as these glands provide the majority of the seminal plasma fraction (Senger, 2003). Additionally, Wickware and colleagues identified the genera *Fusobacterium*, *Histophilus*, *Porphytomonas*, and *Streptobacillus* in association with the epithelial surface of the prepuce and penis of beef bulls (Wickware et al., 2020), some of which are also reported in the semen of bulls (Table 1). Thus, the prepuce- and penis-associated microbes might be another source of the bovine seminal microbiota. Other potential sources such as the circulating blood supply, gut, and mouth are more speculative; however, it has been reported that blood serves as a route of transport for pathogenic bacteria (Jeon et al., 2017), and the gut and oral microbiota may potentially be transported *via* the bloodstream to the reproductive tract (García-Peñarrubia et al., 2020). It is likely that the seminal microbiota is supplied from a combination of these sources (Lundy et al., 2021). A final suggested origin of the seminal microbiota is the transfer of vaginal bacteria to the male reproductive tract during copulation (Mändar et al., 2015; Koedooder et al., 2019a; Farahani et al., 2021). In a recent human study, the seminal and vaginal bacterial communities of sexual partners were reported to share a high number of phylotypes (Mändar et al., 2015). The most abundant genera reported among the shared phylotypes were *Lactobacillus*, *Veillonella*, *Streptococcus*, *Porphyromonas*, and *Atopobium* (Mändar et al., 2015). This indicates that some of the microbial taxa residing within the reproductive tract of natural service breeding bulls is expected to be originated from the vagino-uterine microbiota. Overall, there is a need for topological characterization of the microbial community residing along the urogenital tract of bulls, and microbial sources of bovine seminal microbiota.

Beyond the potential origin of the seminal microbiota, the potential factors that may shape the bovine seminal microbiota composition and community structure include diet, age, sexual activity, and geographical location (Watkins et al., 2018; Sarker et al., 2019; Tomaiuolo et al., 2020). Age is an important factor that may shape the seminal microbiota composition. It has been reported that as a bull ages, the total ejaculate volume as well as the total sperm output increases significantly (Fuerst-Waltl et al., 2006; Snoj et al., 2013). It is reasonable to speculate that these changes in semen parameters may also result in alteration of the composition of seminal microbiota. However, to our best knowledge, the evolution of bovine seminal microbiota by age has not been studied.

In addition to the age, diet could also be an important factor influencing the seminal microbiota. For example, the diet-altered gut microbiota may influence the semen microbial community given the small set of core taxa shared between the human fecal and seminal microbiota (Lundy et al., 2021). The impact of diet on seminal microbiota has been reported in mouse models. Male mice fed high-fat diet (HFD) had altered seminal fluid microbiota characterized by elevated abundance of *Corynebacterium* spp. and reduced *Acinetobacter johnsonii*, *Ammoniphilus* spp., *Bacillus* spp., and *Propionibacterium acnes* compared to the control group which received no HFD (Javurek et al., 2017). While the seminal microbiota was not investigated, the link between the diet and semen quality (Watkins et al., 2018) and the improved sperm quality and spermatogenesis following fecal microbiota transplantation (FMT) in mice indirectly supports that diet could mediate seminal microbiota composition. Given that, in beef cattle production systems in North America where bulls often experience a dramatic shift in their diet composition and dietary intake from grain and forage mix diets during the pre-breeding period to consuming almost exclusively pasture grasses during the breeding season, it is critical to evaluate how bovine seminal microbiota changes in response to dietary change.

Reproductive hormones may also be another factor influencing the composition of the male reproductive microbiota. It has been suggested that the abundance of *Prevotella* is strongly correlated with circulating levels of testosterone (Wang and Xie, 2022). It is, therefore, reasonable to attribute the higher abundance of *Prevotella* in the male reproductive tract compared to that of female reproductive tract to the higher levels of testosterone in men (Haro et al., 2016; Wang and Xie, 2022). Comparably, a microbial shift toward a *Lactobacillus-*dominant community was observed in women during pregnancy, and this shift may be due to the pH change associated with increased estrogen levels (MacIntyre et al., 2015; Koedooder et al., 2019a). In addition, shifts in the gut microbiome of Phayre’s leaf monkeys during different reproductive stages (cycling, pregnancy, and lactation) have been linked to the altered concentrations of reproductive hormones (Mallott et al., 2020). Altogether, this evidence suggests that reproductive hormones could be another factor that can shape bovine seminal microbiota.

An additional factor that may influence bovine seminal microbiota include management factors such as housing and environment, antibiotic exposure, and vaccination. In general, antibiotic treatment disrupts the microbiome of any given niche in animals receiving antibiotics (Koedooder et al., 2019a; Ramirez et al., 2020; Patangia et al., 2022). Housing and environment likely has effect on the seminal microbiota. A significant effect of different flooring systems (e.g., conventional pens with a slatted floor vs. enriched pen floor covered with deep straw) on the fecal microbiota of pigs has been reported (Kubasova et al., 2017). Distinct bacterial communities were identified in different bedding materials, including sand bedding, concrete floor, and compost bedding used in dairy cattle housing (Li et al., 2021a). Thus, changes in the cattle bedding types (straw or wood shavings), and muddier or drier bedding may influence urogenital microbiota of bulls. Also, as observed with different fecal microbiota composition in beef cattle that were raised on pasture vs. feedlot dry lots (Zhang et al., 2021), the microbial composition in the semen of the bulls on the pasture is most likely different from that of the bulls housed in pens. Furthermore, the microbial community composition in reproductive tracts of bulls housed with other bulls might be distinct from bulls housed with cows given the expected greater abundance of microbial species originating from the vagina of cows. The bulls housed in group lots with other bulls, however, are expected to contain some fecal-associated microbial species as the bulls tend to mount each other. This concept is supported by the previous reports of the phyla Firmicutes and Bacteroidetes as abundantly present in bovine fecal microbiota (Hagey et al., 2019; Zhang et al., 2021) and they are also frequently reported in bovine semen and prepuce samples (Table 1). It may be worthwhile for future bovine seminal characterization studies to include samples of fecal and soil or bedding material to further confirm the impact housing plays on the seminal and preputial microbiota.

Finally, environmental temperature may illicit an effect on the microbiota of the internal and external genitalia of bulls in various climates or during seasonal changes (Wickware et al., 2020). While there is currently no direct evidence of this available in the literature, there is evidence of environmental temperatures creating both heat and cold stress in cattle that lead to changes in steroidogenesis, rumen bacterial diversity, and metabolic function (Roth et al., 2001; Tajima et al., 2007; O'Brien et al., 2010; Hu et al., 2021). It has also been shown that certain bacteria can quickly adapt to changes in environmental temperature, altering their metabolism and cell structure as a survival mechanism (Onyango et al., 2013; Alreshidi et al., 2015). The organisms able to adapt in such a manner may hold a consistent presence in the microbiome of animals in climates with drastic seasonal changes while others may not. This is a worthwhile factor that has great potential for future investigation.

## Female reproductive tract microbiome

### Microbial community composition along the female reproductive tract in cattle

Compared to the male reproductive tract, the microbial community residing along the female reproductive tract has been relatively well characterized in cattle (Table 2). Comprehensive discussion of the taxonomic composition of the female reproductive microbiota is not within the scope of the present review, and therefore, readers are referred to recent reviews for additional information regarding the bovine female urogenital microbiota (Ong et al., 2021; Adnane and Chapwanya, 2022; Amat et al., 2022). Briefly, within the bovine female reproductive tract, microbial communities associated with the vagina, cervix, and uterus have been characterized using high-throughput sequencing techniques (Table 2).

**Table 2:** Taxonomic identification of female reproductive tract microbiota from several recent high-throughput sequencing-based studies.

**Species population**	**Country of origin**	**Samples processed for**	**Collected samples**	**Phylum reported**	**Genera reported**	**Key findings**	**References**
20 Crossbred beef cows [1–3 years of age]	United States	16S rRNA gene sequencing (V3-V4); Illumina MiSeq	Vaginal cervico-vagnial lavage	Bacteroidetes, Fusobacteria, and Proteobacteria	*Aggregatibacter*, *Streptobacillus*, *Cronobacter*, *Phoncoenobacter*, *Sediminicola*, and *Sporobacter*	Bacteria dominated samples numerically, but Archaea were detected in 95% of samples. The common order of Archaea identified were Desulfurococcales Lactobacilli were detected in 90% of samples, but at low relative abundance	Swartz et al. (2014)
16 Dairy cows 1–36 days postpartum [Age not reported]	United States	16S rRNA gene sequencing (V4); Illumina MiSeq	Uterine fluid	Bacteroidetes, Firmicutes, Fusobacteria, Proteobacteria, and Tenericutes	Not reported (taxonomic identification was done on the family level)	The uterine bacterial community, regardless of health status, was composed of the main bacterial phyla: Bacteroidetes, Firmicutes, Fusobacteria, Tenericutes, and Proteobacteria The phylogenetic diversity of all samples showed gradual change over the postpartum period	Santos and Bicalho (2012)
97 Primiparous Holstein heifers	United States	16S rRNA gene sequencing (V1-V2); Roche 454 GS-FLX System Titanium Chemistry	Uterus	Firmicutes	*Peptoniphilus*, *Helcococcus*, *Peptostreptococcus*, *Anaerococcus*, *Lactobacillus*, *Anaerovibrio*, *Clostridium*, *Streptococcus*, *Roseburie*, *Oscillibacter*, *Geobacillus*, and *Staphylococcus*	*Bacteroides* spp. and *Ureaplasma* spp. were reported significantly higher in cows suffering from metritis *Geobacillus spp*. were higher in samples from non-metritic cows	Machado et al. (2012)
				Bacteroidetes	*Paludibacter*, *Parabacteroides*, *Prevotella*, *Porphyromonas*, *Alistipes*, and *Bacteroides*		
				Proteobacteria	*Acinetobacter*, *Acidovorax*, *Campylobacter*, *Escherichia/Shigella*, *Sphingopyxis*, *Proteus*, and *Halomonas*		
				Tenericutes	*Mycoplasma*, *Ureaplasma*		
				Fusobacteria	*Streptobacillus*, *Sneathia*, and *Fusobacterium*		
				Actinobacteria	*Arcanobacterium*, *Propionibacterium*		
				Spirochaetes	*Treponema*		
				Other	*Bacillariophyta*, *TM7_genera_incertae_sedis*		
21 Postpartum (5 days) Holstein Cows [age not reported]	Canada	16S rRNA gene sequencing (V4); Illumina MiSeq	Uterus of healthy cows	Firmicutes, Proteobacteria, Bacteroidetes, and Fusobacteria	*Escherichia/Shigella*, *Bacillus*, *Fusobacterium*, *Paracoccus*, *Porphyromonas*, and *Staphylococcus*	The uterine microbiome of cattle with clinical endometritis showed differences in alpha and beta diversity compared to subclinical endometritis cattle and healthy cattle The dominating bacteria cultured from cattle with clinical endometritis was *Trueperella pyogenes* The most abundant bacterial genera’s that resulted from metagenomic sequencing correlated with those that grew in culture	Pascottini et al. (2020)
			Uterus of subclinical endometritis cows	Firmicutes, Proteobacteria, Bacteroidetes, and Fusobacteria	*Porphyromonas*, *Paracoccus*, *Bacillus*, *Fusobacterium*, *Staphylococcus*, and *Bacteroides*		
			Uterus of clinical endometritis cows	Bacteroidetes, Proteobacteria, Firmicutes, and Actinobacteria	*Trueperella*, *Fusobacterium*, *Bacteroides Porphyromonas*, *Collinsella*, and *Paracoccus*		
20 Nellore bovine cows and heifers, pregnant and non-pregnant	Brazil	16S rRNA gene sequencing (Multi-V-region); Illumina MiSeq	Vagina	Firmicutes, Bacteroidetes, and Proteobacteria	*Aeribacillus*, *Bacteroides*, *Clostridium*, *Ruminococcus*, *Rikenella*, *Alistipes*, *Bacillus*, *Eubacterium*, and *Prevotella*	No hormonal influence was observed on microbial diversity Bacterial abundance was reduced while archaeal population increased in pregnant animals Top archaea phylum was reported as Euryarchaeota (genus -*Methanobrevibacter*) Top fungal phylum reported as Ascomycota (genus-*Mycosphaerella*)	Laguardia-Nascimento et al. (2015)
78 Brangus heifers, pregnant and non-pregnant	United States	16S rRNA gene sequencing (V4); Illumina MiSeq	Vagina	Tenericutes	*Ureaplasma*, *Mycoplasma*, and *RF39_unclassified*	Overall bacterial community were not different in heifers with high, medium, or low estradiol concentrations No difference in bacterial composition was identified between pregnant and non-pregnant groups	Messman et al. (2020)
				Proteobacteria	*Histophilus*, *Mannheimia*, *Pasteurella*, *Pasteurellaceae_unclassified*, and *SUP05_ unclassified*		
				Fusobacteria	*Fusobacterium*, *Leptotrichiaceae_unclassified*, *Streptobacillus*,and *Leptotrichia*		
				Firmicutes	*Ruminococcaceae_unclassified*, *Helcoccus*, *Lachnospiraceae_unclassified*, *Oscillospira*, and *SMB53*		
162 Holstein dairy cows	United States	16S rRNA gene sequencing (V4); Illumina MiSeq	Vagina	Proteobacteria, Firmicutes, Bacteroidetes, Fusobacteria, Tenericutes, and Actinobacteria	*Gallibacterium*, *Sneathia*, *Mannheimia*, *Ruminococcus*, *Fusobacterium*, *Porphyromonas*, *Bacteroides*, *Streptococcus*, *Pseudomonas*, *Leptotrishia*, *Clostridium*, *Helococcus*, *Moraxella*, and *Pedobacter*	In this study, the microbial community composition between the dam’s vaginal microbiota and the calf’s upper respiratory tract overlapped by 63% The dam’s vaginal microbiota appears to vertically transmit to the upper respiratory tract of the newborn calf	Lima et al. (2019)
20 Virgin Holstein-Friesian heifers [13–16 months of age]	Spain	16S rRNA gene sequencing (V3-V4); Illumina MiSeq	Vagina	Tenericutes, Firmicutes, Bacteroidetes, Proteobacteria, Actinobacteria, Fusobacteria, Epsilonbacteraeota, and Patescibacteria	*Ureaplasma*, *Histophilus*, *f_corynebacteriaceae*, *Porphyromonas*, *Mycoplasma*, and *Ruminococcaceae UCG-005*	Tenericutes, Firmicutes, and Bacteroides were the most abundant bacterial phyla and comprised over 75% relative abundance Microbial richness variance was significantly higher during the luteal phase samples compared to follicular phase samplesLactobacillus was found at low relative abundance but was more abundant in the follicular phase compared to the luteal phase	Quereda et al. (2020)
40 Holstein dairy cows	China	16S rRNA gene sequencing (V4-V6); Illumina HiSeq	Cervix of healthy cattle	Firmicutes, Proteobacteria, Bacteroidetes, and Fusobacteria	*Ruminococcaceae_UCG-005*, *Fontimonas*, *Pseudomonas*, and *Ruminococcaceae_UCG-010*	The most predominant phyla of the cervix were Firmicutes Bacterial diversity deceased in cattle with Metritis The most predominant genus of the cervix was *Porphyromonas* and *Fusobacterium*	Wang et al. (2018)
			Cervix of Metritis cattle	Bacteroidetes, Fusobacteria, and Firmicutes	*Porphyromonas*, *Fusobacteria*, and *Bacteroides*		
45 Virgin and pregnant Angus-cross heifers [9 months of age]	United States	16S rRNA gene sequencing (V3-V4); NovaSeq 6000	Vagina of virgin heifers	Firmicutes, Bacteriodota, Actinobacteriota, and Proteobacteria	*Corynebacterium*, *Oscillospiraceae UCG-005*, *Rikenellaceae RC9 gut group*, *Bacteroides*, and *Chrisrensenellaceae_R_7_group*	41 OTUs were shared in 60% of all samples from both yearling and virgin heifers, suggesting a core taxa 80% of the core taxa belonged to the phyla Firmicutes	Amat et al. (2021b)
			Vagina of pregnant heifers	Firmicutes, Bacteriodota, Actinobacteriota, and Proteobacteria	*Corynebacterium*, *Oscillospiraceae UCG-005*, *Romboutsia*, *Chrisrensenellaceae_R_7_group*, and *Rikenellaceae RC9 gut group*		
10 Virgin dairy heifers [14 months of age] and five pregnant dairy cows [third trimester]	United States	16S rRNA gene sequencing (V4); Illumina MiSeq	Pregnant Uterus	Firmicutes, Bacteroidetes, and Proteobacteria	Taxonomic identification done on the class level	*A uterine microbiome is present in sexually mature bovines and at conception*, *and is maintained during gestation*	Moore et al. (2017)
			Virgin Uterus	Firmicutes, Bacteroidetes, and Proteobacteria	Taxonomic identification done on the class level	*Minor differences in the microbiome of the amniotic fluid*, *placentome*, *intercotyledonary placenta*, *and cervical lumen were found*	
31 Crossbred or Droughtmaster heifers and cows of various age and reproductive status	Australia	Oxford Nanopore Technologies long-read adaptive sequencing	Vagina of prepubertal females	Ascomycota, Proteobacteria, Firmicutes, Bacteroidetes, and Actinobacteria	Taxonomic identification done on the species level	*Clostridium botulinum* and *Escherischia coli* was a predominant species in females of each reproductive stage but was not indicative of disease Ascomycota was identified as a member of the commensal phyla Breed makeup was determined to be less of an influencer on reproductive metagenome than previously suggested The abundant microorganisms identified in the vaginal samples have the possibility of originating from the soil and feces	Ong et al. (2022)
			Vagina of cycling females	Proteobacteria, Firmicutes, Ascomycota, and Actinobacteria	Taxonomic identification done on the species level		
			Vagina of pregnant females	Proteobacteria, Firmicutes, Ascomycota, and Actinobacteria	Taxonomic identification done on the species level		
			Vagina of postpartum females	Ascomycota, Firmicutes, Proteobacteria, and Bacteroides	Taxonomic identification done on the species level		

Overall, the vaginal bacterial microbiota of cattle is mainly consisting of Firmicutes, Bacteroidetes, Proteobacteria, Fusobacteria, and Tenericutes. Within these bacterial phyla, most frequently identified bacteria genera include *Corynobacterium*, *Oscillospiraceae* UCG-005, and *Ruminococcaceae* UCG-005. *Uroplasma*, *Histophilus*, *Mannheimia*, *Pasteurella*, *Mycoplasma*, *Trueperella*, and *Clostridium* (Table 2). The relative abundance of both phyla and genera may vary depending on the host age, physiological stage (pregnant vs. non-pregnant), and health status (Table 2). In addition to being a harbor for opportunistic pathogens associated with bovine respiratory disease and liver abscesses, the bovine vagina is also colonized by methanogenic archaea (Amat et al., 2021b).

According to the only available study in which cervical microbiota of dairy cows was characterized using 16S rRNA gene sequencing, there is a relatively rich and diverse microbial community colonized in the bovine cervix. Firmicutes (46%) and Proteobacteria (23%) are the most predominant phyla constituting the bovine cervical microbiota of healthy cattle (Wang et al., 2018; Table 2). The major bacterial genera are *Porphyromanas* and *Fusobacterium*. Depending on the different physiological stage and reproductive health state, the cervical microbiota changes in community composition and diversity (Wang et al., 2018).

The uterus has traditionally been considered sterile, as the cervix is believed to serve as a barrier to prevent ascending of bacteria from the lower genital tract into the uterus. However, the application of high-throughput sequencing challenged this dogma, and it is now accepted that the uterine cavity is home to thousands of commensal bacteria and archaea (Chen et al., 2017; Galvão et al., 2019; Toson et al., 2022). The uterine microbiota is present at the time a heifer reaches reproductive maturity and during pregnancy (Moore et al., 2017), and post-partum (Table 2). The uterine microbial community in cattle is composed of the main bacterial phyla Bacteroidetes, Firmicutes, Fusobacteria, Tenericutes, and Proteobacteria (Table 2). Like cervical microbiota, *Fusobacterium* and *Porphyromonas* spp. are abundant within the uterine microbial community along with *Bacteroides*, *Streptococcus*, and *Bacillus* genera (Table 2). *Mycoplasma*, *Ureaplasma*, and *Trueperella* are also frequently found in the bovine uterus experiencing endometritis. Overall, many bacterial taxa are shared among vaginal, cervical, and uterine microbial communities in cattle. Potential sources of the uterine microbiota are believed to be: (1) hematogenous spread of microbes from the gut and oral microbiota; (2) natural ascension of vaginal bacteria to the upper genital tract through the cervix or the introduction of the vaginal bacteria into the uterus *via* assisted reproductive technology-related procedures; (3) transmission of bacteria *via* the seminal microbiota (Altmäe, 2018; Baker et al., 2018). Considering that the seminal microbiota is one of the potential microbial seeders for the female reproductive tract, it is important to understand interactions between the two microbial communities and their subsequent impact on fertilization and pregnancy.

### Evidence suggesting the association of vagino-uterine microbiome with reproductive health and fertility

Emerging lines of evidence derived from human and other vertebrate animal models suggest that microbiomes residing within the female reproductive tract may influence reproductive efficiency (Koedooder et al., 2019a; Tomaiuolo et al., 2020; Rowe et al., 2021). Recent high-throughput sequencing-based studies have identified distinctive vaginal microbiota between fertile and infertile women, and some key bacteria seem to show a species and/or strain specific effect For example, Zhao et al. (2020) observed that women suffering from infertility exhibited a reduced vaginal microbial diversity (the number and relative abundance of microbial taxa) and richness (the number of microbial taxa present in a given environment; Hong et al., 2006) compared with fertile women. Infertile women harbored a higher abundance of *Atopobium*, *Aerococcus*, and *Bifidobacterium* genera, and a lower abundance of *Lactobacillus* and *Leuconostoc* (Zhao et al., 2020). However, Koedooder and colleagues (2019) predicted pregnancy outcome based on the vaginal microbiota composition of 192 women. The model, with relatively high accuracy, was able to predict a subgroup of women who were less likely to become pregnant based on their vaginal microbiota profile. They showed that the abundance of *Lactobacillus crispatus* was negatively associated with fertility and was an important factor in predicting pregnancy. This is interesting, as the genera *Lactobacillus* is generally regarded as a probiotic and is a prominently present in the human vagina; yet there is evidence that the species *Lactobacillus crispatus* in particular has a negative impact on fertility (Li et al., 2021b). Li and colleagues were able to demonstrate that *L*.*crispatus* elicits a negative effect on fertility by adhering to sperm and significantly reducing motility (Li et al., 2021b), which ultimately can decrease the chance of pregnancy. These studies together indicate that the vaginal microbiota is not only associated with vaginal health, but that there is a great need to further research on the role significant bacterial species play. It is highly likely that microbial interactions occurring during coitus and during pregnancy are incredibly influential to reproductive outcomes.

While direct evidence is still lacking, recent developments suggest the potential role the uterine microbiome may have in regulation of endometrial physiology and reproductive health, and thereby influence fertilization and pregnancy outcomes (Baker et al., 2018; Benner et al., 2018). Moreno et al. (2016) reported that dysbiosis of the endometrial microbiota characterized by a non-*Lactobacillus*-dominated microbial community was associated with significant decreases in implantation rate, pregnancy establishment, and live birth weight (Moreno et al., 2016). One of the potential mechanisms through which an altered uterine microbiota exerts a negative effect on the success of implantation is by a microbiota-triggered inflammatory response in the endometrium that ultimately interferes with the adherence of the blastocyst to the endometrial mucosa (Bardos et al., 2019). Galvão and collegues (2019) identified the association between the uterine microbiota and uterine disease in cattle. Dairy cows that had metritis exhibited endometrial microbiome dysbiosis, which was characterized by reduced microbial richness, and an increase in *Bacteroidetes* and *Fusobacteria* abundance (Galvão et al., 2019). Likewise, the uterine microbiota of dairy cows diagnosed with clinical endometritis (CE) differed significantly from that of healthy cows (Pascottini et al., 2020). The uterine microbiota of animals with CE had a decrease in diversity and was dominated by *Trueperella pyogenes*. Similar uterine microbiota dysbiosis was reported in women who suffered from recurrent pregnancy loss (Pillarisetty and Mahdy, 2022). These studies together suggest that the uterine microbiota may play a significant role in reproductive success, and the dysbiosis of this microbiota may compromise conception or implantation and increase pregnancy loss.

### Female reproductive microbiome and its influence beyond reproductive health and fertility

The female reproductive microbiome may not only impact reproductive health and fertility, but it may also extend its influence on offspring development. Strongly debated is the presence of a fetal microbiota. It has long been accepted that the fetal environment is sterile and must remain sterile throughout gestation for the sake of fetal viability, and the fetus only begins to be colonized or ‘seeded’ during and after parturition (Perez-Muñoz et al., 2017; de Goffau et al., 2019). However, recent reports of microbial communities in the placenta and uterus of gestating women and cattle are challenging that narrative (Parnell et al., 2017; Baker et al., 2018; Benner et al., 2018; Guzman et al., 2020; Amat et al., 2021a). It may be likely that the microbial community of the placenta and the uterus “seed” the fetus and provide the “pioneering microbiome” as suggested by Guzman and colleagues (Guzman et al., 2020). This idea originates from the identification of microorganisms in fetal meconium (Jiménez et al., 2008; Moles et al., 2013; Guzman et al., 2015). The presence of microorganisms in meconium—the first stool passed by newborns—would mean that the newborn’s gastrointestinal tract is in fact not sterile but colonized *in utero* by pioneering microbes. It is important to note that the dominant argument against this idea is that the risk of contamination is very high, and early studies have been criticized for their minimal use of negative controls. It is still debated that a healthy placenta harbors no microbiome, and the studies that have identified the presence of bacteria in the fetal environment are dealing with unhealthy tissues (de Goffau et al., 2019).

To address this concern and provide further evidence of the fetal microbiota, a recent study (Guzman et al., 2020) used both culturing and sequencing along with stringent contamination controls to identify microorganisms in ruminal fluid, cecal tissue, cecal fluid, meconium, intestinal tissue, and amniotic fluid of fetal calves at 5, 6, and 7 months of gestation (mid gestation). After ruling out the possibility of contamination from the media, extraction kits, labware, dissection table and tools, ambient air, amniotic sac, mesentery, caecum, and rectum; this study was able to confirm the presence of bacterial and archaeal communities in the fetal gastrointestinal tract (Table 3; Guzman et al., 2020). This may be indicative of microbial selection occurring in these compartments. Even more interesting, this study found that the abundances of both archaeal and bacterial communities increase from 5 to 7 months in gestation, which could indicate a developmental process involved in fetal microbial colonization (Guzman et al., 2020).

**Table 3:** Taxonomic identification of fetal-associated microbiota from several recent high-throughput sequencing-based studies.

**Species population**	**Country of origin**	**Samples processed for**	**Collected samples**	**Phylum reported**	**Genera reported**	**Main findings**	**References**
Angus-cross fetal calves [12 weeks gestation]	United States	16S rRNA gene sequencing (V4); Illumina MiSeq	Allantoic Fluid	Proteobacteria, Firmicutes, and Actinobacteria	*Acidovorax*, *Acinetobacter*, *Stenotrophomonas*, *Brucella*, *Anoxybacillus*, *Sphingomonas*, *Lactobacillus*, and *Allorhizobium-Neorhizobium-Pararhizobium-Rhizobium*	Thirty-nine different bacterial and archaeal phyla were identified among four fetal compartments Four bacterial phyla dominated across all fetal compartments	Amat et al. (2021a)
			Amniotic Fluid	Proteobacteria, Firmicutes, and Actinobacteria	*Acidovorax*, *Acinetobacter*, *Stenotrophomonas*, *Brucella*, *Anoxybacillus*, *Sphingomonas*, *Lactobacillus*, and *Allorhizobium-Neorhizobium-Pararhizobium-Rhizobium*		
			Intestine	Proteobacteria, Firmicutes, and Actinobacteria	*Acidovorax*, *Acinetobacter*, *Stenotrophomonas*, *Brucella*, *Anoxybacillus*, *Sphingomonas*, *Lactobacillus*, and *Allorhizobium-Neorhizobium-Pararhizobium-Rhizobium*		
			Placental Tissue	Proteobacteria, Firmicutes, and Actinobacteria	*Acidovorax*, *Acinetobacter*, *Stenotrophomonas*, *Brucella*, *Anoxybacillus*, *Sphingomonas*, *Lactobacillus*, and *Allorhizobium-Neorhizobium-Pararhizobium-Rhizobium*		
Angus × Friesian fetal calves [5–7-month gestation]	Australia	16S rRNA gene sequencing (V3–V4); Illumina MiSeq	Rumen Fluid	Actinobacteria, Proteobacteria, and Firmicutes	*Corynebacterium*, *Gordonia*, *Propionibacterium*, *Bacillus*, and *Staphylococcus*	All five fetal compartments contained a pioneer microbiome men tissues contained the lowest bacterial and archaeal abundance The dominant bacterial phyla identified in amniotic fluid was different than the dominant phyla in rumen, cecal, and meconium samples	Guzman et al. (2020)
			Rumen Tissue	Actinobacteria, Proteobacteria, and Firmicutes	*Propionibacter*, *Lactococcus*, and *Staphylococcus*		
			Cecal Tissue	Actinobacteria, Firmicutes, and Proteobacteria	*Staohylococcus*, *Tepidiphilus*		
			Cecal Fluid	Firmicutes, Proteobacteria	*Streptococcus*, *Buttiauxella*, *Escherichia/Shigella*, *Obesumbacterium*, and *Acinetobacter*		
			Amniotic fluid	Bacteroidetes, Firmicutes, and Proteobacteria	*Streptococcus*, *Paracoccus*, *Buttiauxella*, and *Stenotrophomonas*		
			Meconium	Actinobacteria, Firmicutes, and Proteobacteria	*Corynebacterium*, *Gordonia*, *Propionibacterium*, *Staphylococcus*, *Anaerococcus*, and *Escherichia/Shigella*		
25 Belgian Blue calves [full term]	Finland	16S rRNA gene sequencing (V3-V4); Illumina MiSeq	Amniotic Fluid	Proteobacteria, Firmicutes, Bacteroidetes, and Actinobacteria	*Staphylococcus*, *Streptococcus*, *Delftia*, *Sphingomonas*, *Enterococcus*, and *Enterococcus*	Both the amniotic fluid and meconium shared similar phyla; individual variation was observed Amniotic fluid had insignificant amounts of bacterial DNA, but the gene sequence profile was different than negative controls, suggesting the presence of microbial DNA but at very low abundance	Husso et al. (2021)
			Meconium	Proteobacteria, Firmicutes, Bacteroidetes, and Actinobacteria	*Delftia*, *Staphylococcus*, *Clostridium sensu stricto 1*, *Acinetobacter*, unclassified *Burkholderiaceae*, and *Corynebacterium 1*		

In addition, our group also identified microbial presence in fetal tissues of beef cattle at 12-week of gestation (Amat et al., 2021a; Table 3). Amniotic and allantoic fluid, and intestinal and placental cotyledon tissues all had microbial DNA representing diverse microbial communities in each of these fetal compartments. In support of sequencing results, scanning electron microscopy imaging also revealed the presence of bacterial cells in fetal fluid (Amat et al., 2021a). The dominant bacterial phyla of these samples were Proteobacteria, Firmicutes, Actinobacteria, and Bacteroidetes, which are also reported as major phyla in mid-gestation and of full-term fetuses (Table 3; Guzman et al., 2020; Husso et al., 2021). We also observed that the microbial community structure differed significantly between allantoic and amniotic fluids and between the fetal intestine and placenta (Amat et al., 2021a). Microbes have also been cultured and imaged from second trimester human fetal tissues (Mishra et al., 2021). Using 16S rRNA sequencing on tissues from fetal skin, gut, placenta, and lung, this study detected sparse yet consistent microbial DNA presence and upon culturing was able to produce microbial colonies in both aerobic and anaerobic conditions. These colonies varied in morphologies and size between tissue types, which indicates variable species between fetal organs (Mishra et al., 2021). In addition to the presence of microbiota in the fetal gut, a recent study reported that human fetal lungs harvested between 11 and 20 weeks of gestation harbored microbial DNA signatures of 48 different taxa (Al Alam et al., 2020). Together, these bovine and human studies suggest the presence of *in utero* microbial colonization, and that the fetal microbiome continues to evolve as gestation progresses.

These findings raise a critical question as to why *in utero* colonization occurs, and to what purpose a fetal microbiome may serve. One of the speculations is that the fetal microbiota may participate in fetal immune programming and thereby prepare the fetus for life outside of the womb. Mishra and colleagues argued that the bacterial signals function to activate and prime fetal memory T cells (Mishra et al., 2021). The fetal T cells have been shown to respond to commensal microbe activation upon pathogen invasion of the mucosal barrier and form a memory (Mishra et al., 2021). After incubating fetal T cells with fetal strains of *Staphylococci* and *Lactobacilli*, the T cells showed significant memory expansions in *Staphlyococci* exposed T-cells and in *Lactobacilli* exposed T cells compared to the controls (Mishra et al., 2021). This implies that bacteria are able to activate memory T cells in the fetus, which suggests that exposure to microbes *in utero* may have a prolonged effect and functions to avoid a pathological immune response once the fetus is exposed to the microbially rich environment outside of the uterus (Bolte et al., 2022).

Other emerging evidence highlights the potential involvement of maternal microbiome in DOHaD (Kimura et al., 2020; Amat et al., 2021a). For example, Kimura and colleagues demonstrated that the maternal gut microbiota is involved in metabolic programming of offspring beginning at the embryonic stage (Kimura et al., 2020). Short-chain fatty acids (SCFAs) derived from the maternal gut microbiota reach the placenta and are transferred to the developing embryos where the SCFA propionate mediates insulin levels and sympathetic nervous system development through G-coupled protein signaling pathways (Kimura et al., 2020). Another mouse study demonstrated that the maternal microbiota during pregnancy modulates programming of fetal nervous system development (Vuong et al., 2020). We observed a subtle change in the vaginal microbiota composition in 9-month old beef heifers in response to the maternal restricted gain during early gestation (Amat et al., 2021b), and that the maternal nutritional regime during gestation may influence the fetal microbiota (Amat et al., 2022). This suggests the potential involvement of bovine maternal microbiome in developmental programming. While the role of maternal reproductive microbiome in fetal programming is yet to be explored, based on the evidence showing the involvement of maternal gut microbiome in DOHaD, it is reasonable to speculate that the maternal reproductive microbiome, particularly the uterine microbiome, may also impart a significant role in fetal programming, given that the fetus is in close proximity to the uterus and the microbial environment within. Thus, future studies should investigate the role of the maternal reproductive microbiome in DOHaD.

As discussed above, the microbial continuum residing along the female reproductive tract may not only influence host fertility, conception, and pregnancy outcome, but may also affect offspring development and health *via* fetal programming as a consequence of *in utero* microbial colonization or the microbial metabolites produced by the reproductive microbiota.

## Interactions between the seminal and vagino-uterine microbiome

As suggested by the recently proposed term “complementary semino-vaginal microbiota” (Mändar et al., 2015), the seminal microbiota may indeed serve as an important medium for transmission of microorganisms from the male reproductive tract to the female reproductive tract (Altmäe, 2018). Several studies demonstrated that bacterial species are shared between female and male urogenital microbial communities (Mändar et al., 2015; Kamińska and Gajecka, 2017; Mändar et al., 2018). The similarity between the bovine seminal and vagino-uterine microbial communities at the phylum and genera level (Tables 1, 2) also highlights the microbial exchange taken place between the male and female reproductive microbiota. Thus, transmission of the microorganisms from semen to the vagina, and then to the uterus, could be one of the factors that influence both vaginal and uterine post-breeding microbiota homeostasis.

**Figure 1:**
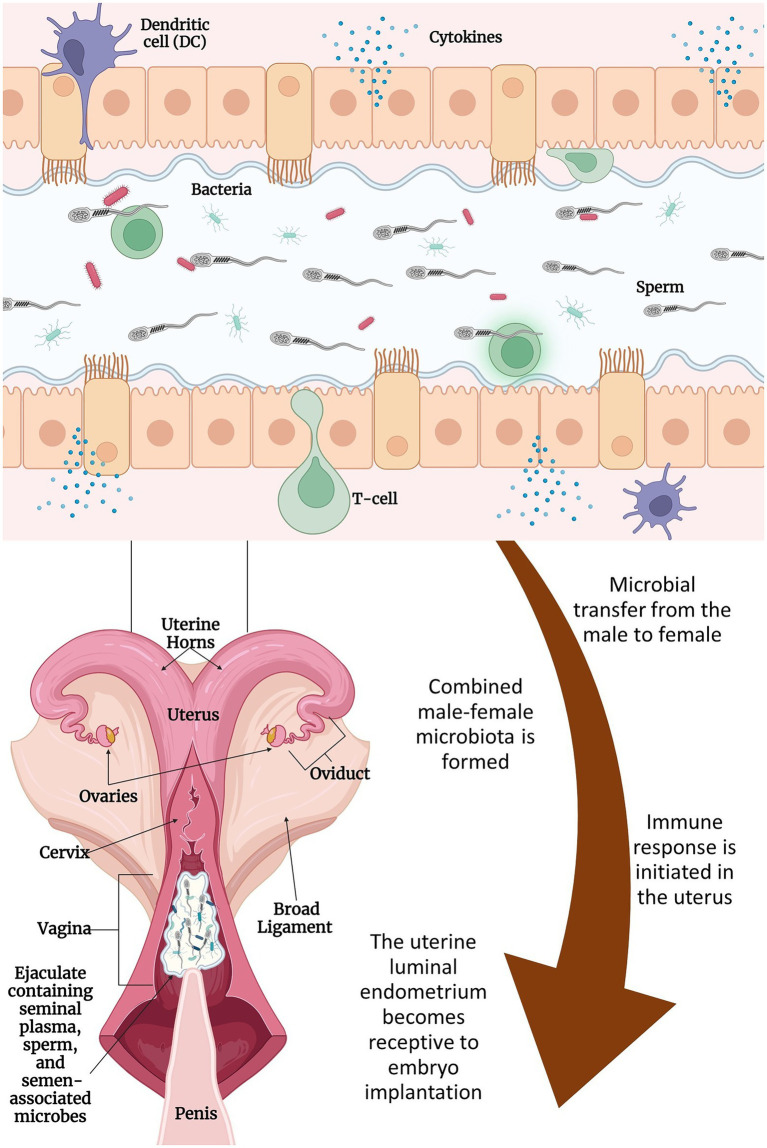
Proposed interactions between seminal and uterine microbiome and their effects on fertilization and implantation: semen is deposited in the cranial vagina of the cow/heifer by the bull. Microbes associated with seminal plasma and sperm mix with microbes associated with the female reproductive tract and migrate through the cervix and into the uterine lumen with the help of contractions from the female’s vaginal and cervical muscularis and the movement of sperm. During this time, the male–female microbes and their metabolites contribute to the immune reaction that takes place in the endometrium of the uterus. Immune cells (e.g., dendritic cells and macrophages) will respond and modulate vascular changes including inflammation of the uterine endometrium. These changes along with other specific responses will lead to the outcome of embryo implantation and contribute to post-conception fetal development. Figure created with BioRender.com.

It has been reported that the seminal microbes entering the vagina could influence resident microbial composition by altering the vaginal pH, which ultimately results in increased abundance of certain bacterial species including *Staphylococci* and *Streptococci* (Borovkova et al., 2011; Koedooder et al., 2019a). Seminal fluid supplies paternal antigens and immune-regulatory factors that can illicit an immune response in the female reproductive tract, thereby imparting an effect on endometrial receptivity, implantation, and subsequent embryo development (Figure 1;Schjenken et al., 2015; Robertson and Sharkey, 2016). This semen-mediated immune response taken place in the female urogenital tract is believed to be attributed mainly to the seminal plasma. Although successful fertilization by sperm cells without seminal plasma can be achieved and is demonstrated by *in vitro* fertilization, both fertility and fetal development are compromised when the seminal plasma is removed. Using rodent models, offspring that were conceived in the absence of seminal plasma showed altered metabolic function and growth (Robertson and Sharkey, 2016). This is thought to be related to the lack of cytokine synthesis in the female reproductive tract, as cytokines further initiate a cascade of specific immune responses that ultimately aid in embryo implantation (Robertson and Sharkey, 2016). Whether or not the microorganisms associated with seminal plasma are involved in this cytokine response has yet to be elucidated. Semen collected for AI in cattle is diluted in an extender before freezing (Ball and Peters, 2004). Depending on the original sperm cell concentrations, 1 ml of ejaculated semen can be diluted up to 65-fold of its volume.^1^ Therefore, the seminal plasma -associated immune response might be less significant in female cattle receiving frozen semen *via* AI. However, the seminal plasma and sperm cell associated microbes that are deposited in the upper reproductive tract of female cattle *via* natural breeding are expected to be involved in immune modulation; and thereby influence the community composition and diversity of the vagino-uterine microbiota. These subjects warrant further research.

### The interactive effects of the seminal and vagino-uterine microbiome on fertilization and subsequent embryo development

Presence of commensal microbial communities in the semen, uterus, and placenta, as well as the recent evidence supporting the *in utero* microbial colonization, together suggest that fertilization and subsequent embryo development are taking place in the presence of microbes. Reports on the presence of *Lactobacillus*-dominant follicular fluid microbiota in women (Pelzer et al., 2011, 2013; Schoenmakers et al., 2019) indicate that oocytes may develop and mature *in vivo* in the presence of follicular fluid microbes (Schoenmakers et al., 2019). Although evidence is lacking, it is highly likely that the male and female gametes may also harbor microbes before conception, and microbes may be present during other reproductive milestones, such as fertilization, cleavage stages of the conceptus, hatching of the blastocyst, and further embryonic development and eventual placentation, fetal growth, and parturition. The questions regarding the source and the role of these microbes in preconception, fertilization, and embryonic development remains to be defined. However, in addition to the uterine microbiota, the seminal microbes that “hitchhike” to the uterus may be part of the microbial community called “temporary combined male and female microbiome,” which presents during the post-coital and preimplantation periods, and can aid conception success (Koedooder et al., 2019a). This combined male and female microbiome may achieve their influence on implantation through enhancing the luminal environment to improve conception rates and mediate the immune response and cytokine production (Figure 1; Robertson and Sharkey, 2016; Schoenmakers et al., 2019; Koedooder et al., 2019a). Therefore, the interactive effects of both the male and female reproductive tract microbiome on fertilization and subsequent embryo development should be investigated, as harnessing the interactive effects of the male and female reproductive tract microbiomes may provide an effective means to enhance reproductive efficiency in cattle.

## Challenges associated with studying the male and female reproductive microbiome in cattle

Studying the reproductive microbiota of both bulls and female cattle can be difficult and present many challenges. First, it is challenging to collect semen samples from intact bulls under complete aseptic condition, and there is likely to be some microorganisms associated with the bull’s skin, the collection chute or mounting dummy, and surrounding air environment present at the time of semen collection. To account for that, it is necessary to collect rigorous contamination control samples, such as ambient air swabs, chute swabs, bull skin swabs, and any other environmental factors that may contribute contamination. It is best practice to sequence these controls and include affiliated data in publications. Second, uterine swabbing is relatively invasive compared to vaginal swabbing, and may present as challenging to collect uterine swab samples from young virgin heifers. Also, characterizing the uterine microbiome during pregnancy is difficult and risky, as uterine swabbing can result in unintentional harm to the placental and fetus. Thus, less invasive uterine sample collection methods should be developed to facilitate sampling from young female cattle and from pregnant cattle during gestation.

Finally, a great challenge emerging in this field is variability in data interpretation that results from multiple methods that can be used to sequence microbial DNA and then taxonomically identify the microbial communities residing within the reproductive tract. While various DNA sequencing methods exist, and have previously been described (Slatko et al., 2018; Waskito et al., 2022), the 16S rRNA gene amplicon sequencing is one that is most frequently used to characterize bovine reproductive microbiota (Ong et al., 2021). The 16S rRNA gene sequencing is a quick and affordable culture-independent method (Gupta et al., 2019) that has considerably advanced the scientific characterizing the microbial communities (Poretsky et al., 2014). However, this method does not come without limitations. The 16S rRNA sequencing targets a single gene using primers specific for a hypervariable region, often the V3-V4 region when studying bacteria (Marotz et al., 2018). There is room to introduce bias through primer selection and through possible unequal PCR amplification of the single targeted gene (Acinas et al., 2005; Poretsky et al., 2014) which may result in over- or under- expression of certain microbes (Ong et al., 2022). Thanks to advancements in bioinformatic tools and reduced costs, there are other sequencing options such as shotgun metagenomic sequencing that does not target a single gene, but rather retrieves the whole genome in short reads (Quince et al., 2017) and allows the capture of not only the taxonomic identification, but also the function of microorganisms. This method also has limitations, especially when used in animal studies. Because this method is untargeted sequencing of the entire sample genome, a majority of the sample will likely include contamination of the host’s genetic material. Methods of reducing host DNA have been published (Marotz et al., 2018; Ganda et al., 2021). This adds expense to a research experiment (Quince et al., 2017) in which the researcher will have to consider when choosing this method of sequencing. Finally, there are methods of long-read sequencing such as, but not limited to, PacBio single-molecule real-time (SMRT) and Oxford Nanopore Technologies (ONT) nanopore sequencing (Amarasinghe et al., 2020). An evaluation of the ONT adaptive sampling method compared to 16S rRNA gene and Shotgun metagenomic sequencing in bovine vaginal samples has recently been published in which the ONT adaptive sampling provided greater percentage of metagenomic data (Ong et al., 2022). Limitations of using long-read sequencing technology include lower accuracy and a high error rate which if not corrected downstream, can create bias as well (Athanasopoulou et al., 2021). The discussion of all the differences in results that may stem from DNA extraction kit selection, to DNA quality, to sequencing platform, and to bioinformatic methods can get very extensive and is beyond the scope of this review. However, it is important for researchers to consider these factors when planning experiments and reviewing other studies.

## Future directions

Although the taxonomic composition of the microbial communities residing within the male and female reproductive tract has been characterized in cattle, the functional features of these microbiomes and their role in defining the reproductive health and male and female fertility are yet to be elucidated. Therefore, there is a need to explore the reproductive microbiome using more targeted, and mechanistic approaches including shotgun metagenomic sequencing, metabolomics, and *in vitro* experimentation. The specific area of research concerning the bovine male reproductive microbiome should be directed toward the following: (1) Discovering the origin of seminal microbiota and factors influencing it; (2) Determining if the seminal microbiota influences sperm metabolism, motility, or morphology; (3) Elucidating the interaction of the semen microbiota with the female reproductive tract and its effects on fertility; (4) Identifying the core seminal microbiota present in healthy individuals; and (5) Further characterization of factors affecting the uterine microbiome and other reproductive microbiomes (e.g., follicular fluid) and their effects on fertility and fetal development. The long-term goal of discovering the role of the reproductive microbiome is to aid in the development of microbiome-targeted strategies such as next-generation probiotics and a synthetic microbial community that can be used along with assisted reproductive technologies to enhance fertility and improve pregnancy in cattle herds. When the claimed role of temporary combined male and female microbiome in fertilization and subsequent embryo development is further supported by *in vitro* and *in vivo* studies, then, the first logical step would be to revisit the current artificial insemination (AI) practice. Bovine semen straws used for AI are stored with antibiotics, which are intended to eliminate all viable bacterial cells in the sperm and seminal fluid. Thus, a new way of storing semen straws without antibiotics might be needed as this will enable the semen associated commensal microbiota to be deposited into the uterus and may ultimately improve pregnancy rates relating to AI. Next, future research should be directed to develop synthetic semino-uterine microbial consortium (SSUMC) based on the seminal and uterine bacterial species that are associated with positive bovine sperm quality and enhanced fertilization. *In-utero* inoculation of the SSUMC before or along with AI may enable microbiome-mediated fertilization and pregnancy outcome improvement. Current bovine AI practice with a rigid quality and microbial contamination control protocol, established use of liquid nitrogen for storage, and mechanisms of intrauterine placement is well suited to store and deliver synthetic microbial consortium into the female reproductive tract in cattle.

In terms of the reproductive microbiome in female cattle, many fundamental questions regarding the vagino-uterine microbiome and its interaction with the seminal microbiota, its involvement in fertility, pregnancy, and developmental programming remain to be understood. To harness the female reproductive microbiome-mediated fertility, future studies should focus on the following areas: (1) Identification of the healthy vaginal and uterine microbiota associated with a positive pregnancy outcome; (2) Understanding of the role of vagino-uterine microbiota in maintaining reproductive health and fertility; (3) Characterizing the interaction of vagino-uterine microbiome with seminal microbiome and its involvement in shaping the respective male and female urogenital microbiota; and (4) Defining the impact of the maternal reproductive microbiota during pregnancy on fetal programming and early life microbiome development.

Given that female fertility and pregnancy outcomes may be influenced by the seminal and uterine microbiome, and interactions between the two microbial communities, it is necessary to apply a holistic approach and consider both male and female reproductive microbiome in future research aiming to develop microbiome-targeted strategies for improved fertility in cattle.

## Conclusion

The microbial communities residing within the male and female reproductive tract are important in reproductive health in cattle. Female fertility and pregnancy outcomes may be influenced not only by its own reproductive tract microbiota but also by the semen-associated microbes that enter the uterus, and the interactions between the seminal and uterine microbiome. In addition to influencing reproductive performance, the impact of the female reproductive tract microbiome seems to extend beyond the pre-conception and early embryonic development stages and may have paramount impact on fetal and offspring development and health, considering the involvement of microbiome in DOHaD and existence of *in utero* fetal microbial colonization. Thus, the microbiome of the male and female reproductive tract holds tremendous potential to improve bovine reproductive health, fertility, and offspring development. Developing strategies to improve fertility and reproductive efficiency in beef cattle by harnessing the seminal and vagino-uterine microbiome and their interaction has important implications for the modern cattle industry in an era defined by increasing scrutiny of antimicrobial use, and genetic selection and management refinement may have reached a plateau.

## Author contributions

SL and SA: conceptualizing and outlining the review. SL and EW: conducted the literature search. SL and SA: drafting the initial manuscript. SL, EW, CD, LR, and SA: manuscript writing, revision, editing, and finalizing. All authors contributed to the article and approved the submitted version.

## Funding

The work presented in this study was financially supported in part by the North Dakota Agricultural Experiment Station as part of a start-up package for SA.

## Conflict of interest

The authors declare that the research was conducted in the absence of any commercial or financial relationships that could be construed as a potential conflict of interest.

## Publisher’s note

All claims expressed in this article are solely those of the authors and do not necessarily represent those of their affiliated organizations, or those of the publisher, the editors and the reviewers. Any product that may be evaluated in this article, or claim that may be made by its manufacturer, is not guaranteed or endorsed by the publisher.

## Footnotes

^1^ https://blog.isperm.co/semen-dilution-and-preservation-in-artificial-insemination/
